# Disseminated melioidosis with sternoclavicular joint abscesses

**DOI:** 10.1590/0037-8682-0033-2023

**Published:** 2023-03-27

**Authors:** Chee Yik Chang

**Affiliations:** 1 Hospital Sungai Buloh, Medical Department, Selangor, Malaysia.

**FIGURE 1:**
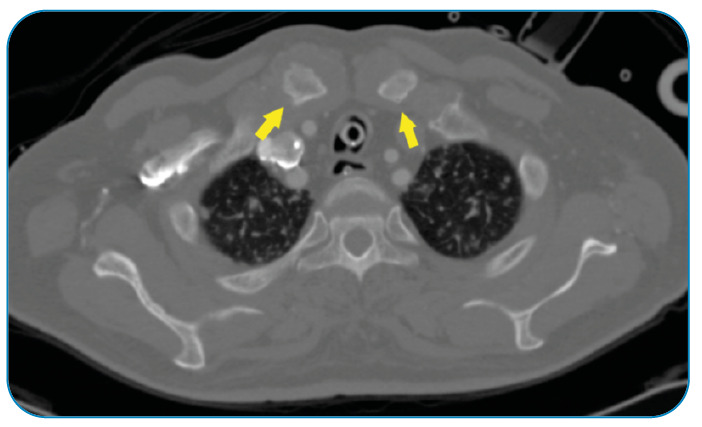
Computed tomography of the thorax showing bilateral sternoclavicular joint abscesses.

A 62-year-old farmer with diabetes mellitus presented with a one-week history of fever, breathlessness, and swelling over the upper chest wall. At initial presentation, the patient was hypotensive and experienced severe respiratory distress, necessitating endotracheal intubation. Physical examination revealed crackles in both lung fields and tender swelling over both the sternoclavicular joints. Chest computed tomography (CT) revealed lung consolidations and soft tissue lesions involving the upper sternum and bilateral sternoclavicular joints, indicating abscesses (Figure 1). *Burkholderia pseudomallei* was isolated from blood and abscess fluid cultures and was found to be susceptible to ceftazidime, imipenem, and trimethoprim-sulfamethoxazole. The patient was administered intravenous meropenem (2 g every 8 h), but his condition did not improve, and he died two weeks later from disseminated melioidosis with multiorgan failure.

Melioidosis is caused by the gram-negative bacterium*Burkholderia pseudomallei*, which is endemic to northern Australia and southeast Asia. Melioidosis can present with diverse clinical manifestations, including pneumonia; skin and soft tissue infection; genitourinary infection; visceral abscesses; and ocular, neurological, and musculoskeletal melioidosis^1^. The latter is uncommon, even in endemic areas, and can manifest as osteomyelitis, septic arthritis, or soft-tissue abscesses. Few cases of melioidosis involving the sternoclavicular joints have been reported^2^ ^,^ ^3^. The treatment of musculoskeletal melioidosis includes antibiotics (intensive phase: meropenem and ceftazidime; eradication phase: trimethoprim-sulfamethoxazole) and surgical intervention in appropriate settings.
